# Prevalence and socio-demographic factors associated with anaemia in pregnancy in a primary health centre in Rivers State, Nigeria

**DOI:** 10.4102/phcfm.v4i1.328

**Published:** 2012-06-14

**Authors:** Geraldine U. Ndukwu, Paul O. Dienye

**Affiliations:** 1 Department of Family Medicine, University of Port Harcourt Teaching Hospital, Nigeria

## Abstract

**Background:**

Anaemia, though a common problem in Nigeria, has not been adequately studied amongst pregnant women in primary health care facilities.

**Objective:**

This study is aimed at determining the prevalence of anaemia and socio-demographic factors associated with anaemia in pregnancy in a primary health centre in Rivers State, Nigeria.

**Methodology:**

This is a cross-sectional study carried out in a primary health centre. Association between variables was analysed using the Chi-square test.

**Results:**

Two hundred and twenty-seven pregnant women whose ages ranged from 16 to 40 years with a mean age of 26.8 ± 4.3 years were recruited for the study. The haemoglobin concentration ranged from 6 g/dL – 15 g/dL with a mean of 10.10 ± 1.27g/dL. A total of 142 (62.6%) participants were anaemic. Anaemia was observed to be least prevalent in women within the extremes of reproductive age (≤ 20 years and 36–40 years). There was no statistically significant association between age, educational level and marital status (*p* > 0.05). The association of anaemia with social class was statistically significant (*p* = 0.000). Severe anaemia was not a common finding amongst the patients but it was significantly associated with educational status (*p* = 0.02) and socio-economic status (*p* = 0.03).

**Conclusion:**

The prevalence of anaemia amongst the pregnant participants in the primary health centre was high. Out of all the socio-demographic characteristics, only socio-economic status was significantly associated with anaemia. It is recommended that the socio-economic status of women should be enhanced in line with the Millennium Development Goals to prevent anaemia and to enhance pregnancy outcomes.

## Abstrait

**Prévalence et facteurs sociodémographiques associés à l'anémie au cours de la grossesse dans un centre de santé primaire dans l'Etat de Rivers, au Nigeria**

**Présentation:**

Bien que l'anémie soit un problème courant au Nigeria, elle n'a pas été étudiée de manière adéquate chez les femmes enceintes accueillies dans des structures de soins de santé primaires.

**Objectif:**

L'objectif de cette étude est de déterminer la prévalence et les facteurs sociodémographiques associés à l'anémie au cours de la grossesse dans un centre de santé primaire dans l'Etat de Rivers, au Nigeria.

**Méthodologie:**

Cette étude consiste en une étude transversale réalisée dans un centre de santé primaire. L'association entre les variables a été analysée à l'aide du test de distribution du khi carré.

**Résultats:**

Deux-cent vingt-sept femmes enceintes âgées de 16 à 40 ans, d'une moyenne d’âge de 26.8 ± 4,3 ans, ont été recrutées pour cette étude. Le taux d'hémoglobine variait entre 6 g/dL et 15 g/dL, avec une moyenne de 10.10 ± 1.27 g/dL. Au total, 142 (62.6%) participants étaient anémiques. L'anémie était moins frequente chez les femmes situées aux extrêmes de l’âge de procréation (≤ 20 ans et 36 à 40 ans). Aucune association statistiquement signicative n'a été observée entre l’âge, le niveau d’éducation et la situation familiale (*p* > 0.05). L'association entre l'anémie et la catégorie sociale était statistiquement significative (*p* = 0.000). L'anémie sévère n’était pas courante chez les patientes, mais était significativement associée au niveau d’éducation (*p* = 0.02) et au statut socioéconomique (*p* = 0.03).

**Conclusion:**

La prévalence de l'anémie chez les participantes enceintes dans le centre de santé primaire était élevée. Parmi les caractéristiques sociodémographiques, seul le statut socioéconomique était significativement associé à l'anémie. Il est recommandé que le statut socioéconomique des femmes soit amélioré conformément aux Objectifs du Millénaire pour le développement afin de prévenir l'anémie et d'améliorer les chances de mener la grossesse à terme et de donner naissance à un bébé en bonne santé.

## Introduction

Anaemia in pregnancy has continued to be a global problem associated with increased maternal morbidity and mortality particularly in developing countries such as Nigeria.^1^ Anaemia is defined as a reduction in the red cell mass in blood resulting in a drop in the amount of oxygen supply to meet the metabolic needs of the body.^2^

### Key focus

Worldwide 41.8% of pregnant women are anaemic as compared with 30.2% non-pregnant women; the most severely affected areas are South-East Asia (48.2%) and Africa (57.1%).^1^ A large proportion of the 17.2 million anaemic pregnant women in Africa live in the west African sub-region.^1^ The prevalence rate in some of the countries range from 50.2% in Togo, 66.7% in Nigeria, 68.3% in Burkina Faso, 72.7% in Benin and 75.1% in Gambia.^1^ Local prevalence studies from Nigeria range from 35.3% in Lagos to 72.0% in Kano State.^3, 4^

In developing countries like Nigeria the cause of anaemia is multi-factorial and varies greatly by geographical location, season and dietary intake.^5, 6^ The most common causes of anaemia in Nigeria include nutritional deficiencies of iron and folate, parasitic diseases such as malaria and hookworm, haemoglobinopathies such as sickle cell disease and recently human immunodeficiency virus infection.^5^ Most of these causes of anaemia in pregnancy are preventable. However, despite the use of iron and folate supplementation and anti-malarial prophylaxis, which are prescribed for pregnant women in ante-natal clinics for the prevention of anaemia, the prevalence of anaemia is still high in the country. This shows that there are other likely underlying factors that are contributing to the high prevalence of anaemia recorded in Nigeria.

An important factor that is common in the tropics but is often overlooked is socio-economic deprivation. This has been linked with the development, severity and outcome of many medical conditions.^3, 7^ Poverty and low standards of living are still major problems facing most developing countries. Regardless of the huge deposits of mineral resources, Nigeria still ranks the 13th poorest country in the world with about 72% of the country's population living below the poverty line (i.e. at $1 per day).^8^ The ability of women to command resources and make independent decisions about their fertility, their health and healthcare also has an impact on maternal anaemia. Where women are afforded a low status in society their health needs are often neglected, and existing health facilities may not be accessed by women in need. In addition, lack of education and understanding about health related issues can contribute to delays in seeking care when it is needed or to the inappropriate management of life threatening pregnancy complications.

Maternal anaemia results in morbidity and mortality in both the mother and the unborn child.^1^ According to the World Health Organization (WHO) the estimates of the global burden of deaths that is attributable to anaemia in women of reproductive age ranges from 16 800 to 28 000 annually with a greater risk of anaemia-related death in younger women.^9^ In Nigeria studies have shown that anaemia contributes to the increased maternal mortality recorded in the nation.^10, 11^ This ranges from 14.6% in North- Central Nigeria to 20% in the western region of the country.^10, 11^ Aanaemia is estimated to contribute to 591 000 perinatal deaths globally per year.^12^

Across the globe, the concern is generally targeted towards the reduction of maternal mortality. In 1987, the World Bank in collaboration with the WHO, the United Nations Fund for Population Activities (UNFPA) and leaders from 45 nations launched the Safe Motherhood Initiative. One of the key components of this initiative is the eradication of anaemia during pregnancy.^13^ It was against this backdrop that in 1989 the African Regional Consultation on the Control of Anaemia in Pregnancy met in Brazzaville in the Democratic Republic of the Congo (DRC), and recommended that studies of prevalence and aetiology of anaemia in pregnancy should be carried out in each of the sub-regions of Africa.^14^ These strategies, it was hoped, would help in the management and control of anaemia amongst pregnant women and reducing maternal mortality.

#### Summary of the key focus

Anaemia in pregnancy has a high prevalence worldwide, especially in developing countries such as Nigeria. Local prevalence figures from Nigeria ranges from 35.3% in Lagos to 72.0% in Kano State. In developing countries such as Nigeria, the most common causes are preventable and include nutritional deficiencies of iron and folate, parasitic diseases such as malaria and hookworm, haemoglobinopathies and recently human immunodeficiency virus infection.

The high prevalence of anaemia in pregnancy despite the use of iron and folate supplementation and anti-malarial prophylaxis shows that there are other likely underlying factors that are contributing to the high prevalence of anaemia recorded in Nigeria. Socio-economic deprivation which has been linked with the development, severity and outcome of many medical conditions is an overlooked factor. Maternal anaemia results in morbidity and mortality in both the mother and the unborn child. Strategies have been put in place by the WHO to control anaemia in pregnancy and reduce maternal mortality.

#### Significance of the study

Published studies on the prevalence and aetiology of anaemia in pregnancy in primary care level is lacking in most parts of Nigeria, especially in the area of the current study. Most of the published studies are from secondary and tertiary health care facilities. This is obviously an obstacle to equitable distribution of resources targeted towards improving maternal health at the different levels of health care. Information from literature indicated that very few published studies in Nigeria have addressed the role of socio-demographic factors on anaemia in pregnancy.^3, 7, 15^ The current study was therefore carried out to determine the prevalence and socio-demographic factors associated with anaemia in pregnant women at booking in a primary care setting in a primary health centre in Rivers State of Nigeria. The study is intended to provide useful information that would help in identifying likely areas for specific intervention for enhanced reproductive health performance.

## Ethical consideration

Approval for this study was obtained from the Ethical Committee of College of Health Sciences and Technology, Port Harcourt before the commencement. The details of the study were thoroughly explained to all the women enrolled for ante-natal care during the health talk session at the clinic. Informed written consent of the participants was obtained before involving them in the study.

## Methods

### Participants

All pregnant women visiting the ante-natal clinic for the first time (i.e. the booking clinic where enrolment of patients for ante-natal care takes place) within the six month period in which this study was carried out were involved in the study.

### Setting

The primary health centre is located within the premises of the Rivers State College of Health Science and Technology, KM 6 Ikwerre Road, Rumueme, Port Harcourt, Nigeria; Port Harcourt is the capital of Rivers State. It is located in the South geopolitical zone of Nigeria along the Bonny River.^16^ All the pregnant women who signed up for ante-natal care in the clinic were asked to undergo laboratory investigations such as haemoglobin, haemoglobin genotype, malaria smear, retroviral screening, stool examination, urinalysis, and venereal disease research laboratory (VDRL) in an attempt to screen them for some common disease conditions, such as anaemia, haemoglobinopathy, malaria, HIV, helminthiasis, urinary tract infection and syphilis.

### Design

This is a cross-sectional, hospital-based study carried out in a primary health centre.

### Sample size

Considering that there was no known study on the prevalence of anaemia amongst pregnant women in a primary health centre conducted in Rivers State, it was assumed that about 50% of pregnant women in the area were anaemic. It was therefore calculated that a minimum sample size of 100 pregnant women will provide an estimate of prevalence with 10% error within 95% confidence limits. However, this minimum sample size was increased to 236 participants to improve the precision of the study.

### Patients’ selection

All apparently healthy pregnant women attending the ante-natal clinic in the primary health centre were enrolled in the study. Only patients who gave their consent whilst enrolling for ante-natal care for the index pregnancy were admitted into the study. All pregnant women suffering from malaria, sickle cell disease, hookworm infestation and HIV at the time of their admission to the study were excluded from participating in the study.

### Procedure

Data was collected from patients by means of pretested semi-structured questionnaires administered by the researchers. Their names were not required on the questionnaire and each subject was assured that the information given was solely for scientific purposes and would be kept confidential. Information on the questionnaire included socio-demographic characteristics such as age, occupation and educational status. The information from the questionnaires was used to group the pregnant women into their different social classes. Education, occupational status and income are the most widely used indicators of socio-economic status (SES). However, in this study the socio-economic classes of the pregnant women were determined using the educational hierarchy developed by Oyedeji:^17^

- Social Class I was awarded to university graduates or its equivalents
- Social Class II was awarded to secondary school certificate holders who also had teaching or other professional training.
- Social Class III was awarded to school certificate or grade II teachers’ certificate holders or its equivalents.
- Social Class IV was awarded to those with Junior Secondary School Class 3 (JSS3) or primary six certificates.
- Social Class V was awarded to those who could neither read nor write, or were considered as illiterate.

Social classes I and II represent the upper class, class III represents the middle class, and classes IV and V represent the lower class.

All the pregnant women were clinically examined after the samples were taken from them for the routine medical tests. The haemoglobin estimation was done using a HemoCue^®^ B-Hemoglobin system (HemoCue AB, Ängelholm, Sweden); this and the cyanmethaemoglobin method are the two methods recommended for use in surveys to determine the population prevalence of anaemia by haemoglobinometry. The HemoCue system consists of a portable, battery-operated photometer and a supply of treated disposable cuvettes in which blood is collected. It gives satisfactory accuracy and precision when evaluated against standard laboratory methods. Long-term field experience has also shown the instrument to be stable and durable with a sensitivity of 82.4% and specificity of 94.2%.^18, 19^ Anaemia in this study is defined by using the WHO criteria of haemoglobin values of less than 11g/dL:^20^

- mild anaemia 9.0–10.9 g/dL,
- moderate anaemia 7.0–8.9 g/dL
- and severe anaemia < 7.0 g/dL.

Thereafter, and depending on the severity of the anaemia, the women were either given haematinics to build up their blood level or they were referred to the obstetrician for appropriate management.

### Analysis

Responses to questionnaires and levels of haemoglobin were coded and entered into a database using SPSS version 17.0 for analysis. Frequency tables were constructed; percentages were calculated and a Chi-square was also calculated to test the association between anaemia and socio-demographic factors. Statistical significance was set at 95% confidence level or at *p*-value of less than or equal to 0.05 (*p*-value ≤ 0.05).

### Results

**FIGURE 1:**
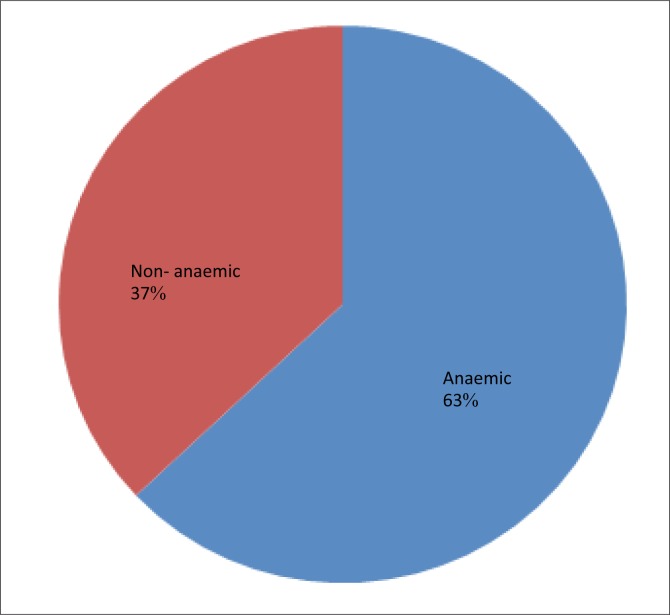
Prevalence of anaemia in pregnancy.

**FIGURE 2:**
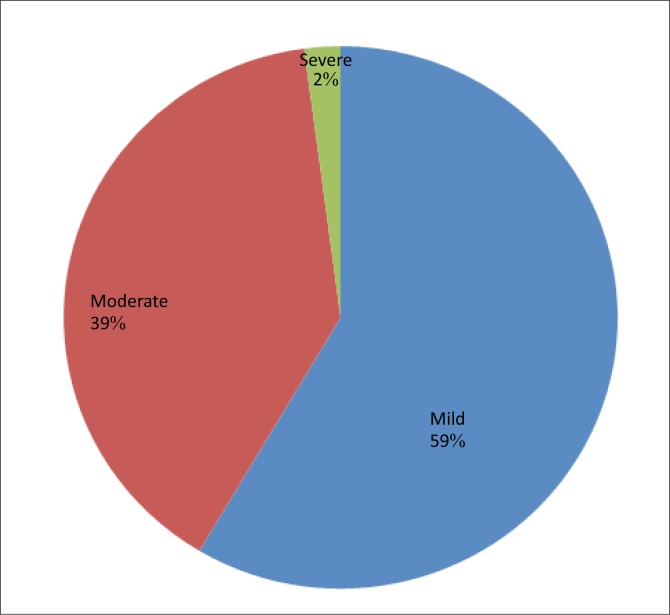
Severity of anaemia in pregnancy in the primary health centre.

Two hundred and thirty six (236) pregnant women were recruited for the study out of which 9 women (3.81%) did not consent to participate in the study. Two hundred and twenty-seven women (96.19%) consented to the stipulations and were involved in the study. The ages of the participants ranged from 16 to 40 years with a mean age of 26.8 ± 4.3 years. Most participants had a secondary-level education and were from the socio-economic class IV (Table 1). The haemoglobin concentration ranged from 615g/dL to 15g/dL with a mean of 10.10 ± 1.27g/dL. A total of 142 (62.6%) participants were anaemic in the current study (Figure 1). Anaemia was observed to be least prevalent in women within the extremes of reproductive age (≤ 20 years and 36–40 years). The association between age and anaemia in pregnancy was, however, not statistically significant (*χ*^2^ = 2.54, *p* = 0.64). The prevalence of anaemia was highest amongst participants with a secondary-level education (58.45%). This relationship between the participants’ level of education and the prevalence of anaemia was not statistically significant (*χ*^2^ = 2.03, *p* = 0.57). Anaemia was also found to be more prevalent amongst married mothers (97.18%) than amongst single women (2.82%), but this relationship was not statistically significant. (*χ*^2^ = 0.12, *p* = 0.73). A direct relationship was found to exist between the prevalence of anaemia and socio-economic class at the primary health centre. This relationship was statistically significant (*χ*^2^ = 42.74, *p* = 0.000). (Table 2). Only 2% of the anaemic participants were severely anaemic (Figure 2). The severity of anaemia was significantly associated with educational status (*p* = 0.02) and socio-economic status (*p* = 0.03) (Table 3).

**TABLE 1:** Socio-demographic characteristics of study population.

Variables	*n*	%
**Age groups (years)**		
16–20	13	5.7
21–25	76	33.5
26–30	96	42.3
31–35	37	16.3
36–40	5	2.2
**Marital Status**		
married	222	97.8
single	5	2.20
**Educational status**		
Tertiary	77	33.9
Secondary	127	55.9
Primary	21	9.3
Non-formal	2	0.9
**Social classification of participants**		
Social class I	4	1.8
Social class II	27	11.9
Social class III	72	31.7
Social class IV	95	41.9
Social class V	29	12.8

*Source*: Authors’ own.

*n*, Given as means of number.

**TABLE 2:** Association between socio-demographic factors and anaemia in pregnancy.

Variables	Anaemic		Non-anaemic		*χ*^2^	*p*-value
	
	*N* = 142	%	*N* = 85	%		
**Ages (years)**						
16-20	11	7.75	2	2.35	*2.54	0.64
21-25	47	33.10	29	34.12	-	-
26-30	56	39.44	40	47.06	-	-
31-35	24	16.90	13	15.29	-	
36-40	4	2.82	1	1.18	-	-
**Educational status**						
Tertiary	43	30.28	34	40.00	*2.03	0.57
Secondary	83	58.45	44	51.76	-	-
Primary	15	10.56	6	7.06	-	-
Non-formal	1	0.70	1	1.18	-	-
Marital status						
Single	4	2.82	1	1.18	*0.12	0.73
Married	138	97.18	84	98.82	-	-
**Socio-economic class**						
Social class I	1	0.07	3	3.53	*42.74	0.000
Social class II	9	6.33	18	21.18	-	-
Social class III	30	21.13	42	49.41	-	-
Social class IV	75	52.82	20	23.53	-	-
Social class V	27	19.01	2	2.35	-	-

* with Yate's correction.

*N*, Given as number of participants.

*χ*^2^, Chi-square.

Social Class I was awarded to university graduates or its equivalents; Social Class II was awarded to secondary school certificate holders who also had teaching or other professional training; Social Class III was awarded to school certificate or grade II teachers’ certificate holders or its equivalents; Social Class IV was awarded to those with Junior Secondary School Class 3 (JSS3) or primary six certificates; Social Class V was awarded to those who could neither read nor write, or were considered as illiterate.

**TABLE 3:** Association of socio-demographic factors and severity of anaemia in pregnancy.

Variables	Mild		Moderate		Severe		*χ*^2^	*p*-value
		
	*n* = 83	%	*n* = 56	%	*n* = 3	%		
**Ages (years)**								
16–20	5	6.02	6	10.71	0	0.00	*4.69	0.79
21–25	29	34.94	20	35.71	0	0.00	-	-
26–30	33	39.76	20	35.71	3	100.0	-	-
31–35	13	15.66	9	16.07	0	0.00	-	-
36–40		3.61	1	1.79	0	0.00	-	-
**Marital status**								
Single	0	0.00	4	7.14	0	0.00	*5.82	0.06
Married	83	100.0	52	92.86	3	100.0	-	-
**Educational status**								
Tertiary	22	26.51	18	32.14	1	33.33	*14.86	0.02
Secondary	55	66.27	29	51.79	2	66.67	-	-
Primary	5	6.02	9	16.07	0	0.00	-	-
Non-formal	1	1.20	0	0.00	0	0.00	-	-
**Socio-economic class**								
Social class I	1	1.20	0	00.00	0	0.00	*16.86	0.03
Social class II	3	3.61	3	5.36)	0	0.00	-	-
Social class III	17	20.48	12	21.43	1	33.33	-	-
Social class IV	51	61.45	25	44.64	2	66.67	-	-
Social class V	11	13.25	16	28.57	0	0.00	-	-

* with Yate's correction.

*N*, Given as number of participants.

*χ*^2^, Chi-square.

Social Class I was awarded to university graduates or its equivalents; Social Class II was awarded to secondary school certificate holders who also had teaching or other professional training; Social Class III was awarded to school certificate or grade II teachers’ certificate holders or its equivalents; Social Class IV was awarded to those with Junior Secondary School Class 3 (JSS3) or primary six certificates; Social Class V was awarded to those who could neither read nor write, or were considered as illiterate.

## Discussion

Very few studies have been carried out in Nigeria focusing on the prevalence of anaemia in pregnancy amongst women registered at the primary health centre. Most studies were either conducted at specialist or general hospitals.

The prevalence rate for anaemia amongst pregnant women admitted to this study was found to be 62.6%. This is high compared with what was obtained in similar studies conducted in different areas in Oyo State (32.8%) and Kano (48.1%) which are both in Nigeria, and in South Africa (57.3%).^15, 21, 22^

The observed lower prevalence rate in Oyo state could be attributed to the fact that it was conducted amongst pregnant women in the ante-natal clinic during their third trimester. These women were likely to be on haematinics which might have improved their haemoglobin levels whereas in this study the haemoglobin level during enrolment for ante-natal care was used, the trimester of pregnancy was not taken into consideration. The difference between findings in this study and the South African study could have emanated from population differences and the retrospective design of their study. They also used the direct cyanmethaemoglobin method in measuring the haemoglobin concentration of their patients as compared to the HemoCue system used in this study. In the Kano study, the screening was done using packed cell volume. Furthermore, considering the fact that differences in methods used in assessing haemoglobin concentration influence the figures obtained, the differences in the prevalence of anaemia is expected.^23^ The comparatively smaller sample size in the present study could also have contributed to the disparity in the prevalence figures.

This study has shown that anaemia is significantly associated with socio-economic status. The prevalence of anaemia in this study was observed to be increasing as the socio-economic status reduced; anaemia occurred more frequently in the lower social classes (IV and V), followed by the middle class (III), and then the upper social class (I and II) where it was lowest. This observation is similar to that reported in similar studies elsewhere.^3, 7^ This is not surprising considering the fact that women in low socio-economic classes are likely to be poorly educated and often have financial constraints. These women cannot afford good health services or they might not have access to health services. The result is that they suffer the deleterious effects of poor nutrition, malaria, HIV, chronic infections and worm infestations.^3^

It has also been shown that the cause of maternal anaemia often has its roots in a woman's life before pregnancy, during infancy or even before a woman's birth, when deficiencies in calcium, vitamins A and D and iron appear for the first time.^24^ Women in the low socio-economic class may therefore have chronic iron deficiency anaemia even before pregnancy; this may be aggravated by the demands of the fetus during pregnancy.

The severity of anaemia in this study was observed to be significantly associated with educational status and socio-economic class. The low incidence of severe anaemia recorded in this study is similar to the 2% reported in Ilesha but lower than 6.7% in South Nigeria.^25, 26^ Because malaria is known as a cause of severe anaemia in pregnancy,^27^ this low incidence of severe anaemia may be a result of successful malaria eradication projects undertaken in the past, including the WHO programme referred to as ‘roll back malaria’ that is an ongoing project in Nigeria. The level of community awareness for the need to treat malaria both in pregnancy and in the pre-pregnant state across Nigeria may therefore be high. The prevalence of severe anaemia in this study amongst women with secondary education is similar to findings in a study by Gautam et al. in India.^28^ This appears to be at variance with other studies in which it is expected to be more in the lesser educated group. It is believed that the small sample size of the study could have accounted for this. Severe anaemia found amongst pregnant women in the middle- and low socio-economic classes is not unexpected in this study. This is due to the fact that the women may likely be unemployed and therefore cannot afford to enrol for ante-natal care, eat nourishing food and/or prevent possible infection. A number of researchers have reported that the prevalence of iron deficiency anaemia and anaemia caused by malaria is higher amongst people living in chronic poverty.^6, 29, 30^ Iron deficiency occurs in families, not because of genetic factors but possibly as a result of economic issues. If an iron-deficient child is identified, the mother and siblings of that child are frequently also deficient. The increased prevalence of anaemia amongst the economically deprived people and people in developing countries is explained in part by the fact that heme iron is almost totally absent from their diets. Also because of economic reasons and food taboos, the diet of pregnant women in many developing countries is effectively almost vegetarian in nature. This leads to nutritional deficiencies such as iron and vitamin B_12_ deficiency. It is also important to realise that severe anaemia is associated with very poor overall socio-economic and health conditions in developing countries such as Nigeria^29, 30^ It is important to note that even in developed countries anaemia during pregnancy can reflect low socio-economic status.^29, 30^ These observations support the claim that socio-economic deprivation is an important factor that predisposes pregnant women to anaemia in most tropical regions including Nigeria. ^3, 7, 26^

Some questions warranting further research remain including ‘is there any difference in the prevalence of anaemia in the primary health centre and tertiary health centre?’ and ‘what is the effect of anaemia on maternal mortality rate in the health centre?’

## Limitations of the study

This study has some limitations: in order to recruit an adequate sample size within the planned study period, a non-probability sampling technique was used. This could have introduced some bias in the study. The study was conducted in a primary health centre in an urban area; this means that the results cannot be extrapolated to a rural population. The actual diet of the participants was not investigated. The role played by dietary deficiencies in the etiology of anaemia therefore remains a subject for further study. It is important to note that this is the only known study on anaemia amongst pregnant women in a primary health centre in South Nigeria.

## Conclusion and recommendations

The findings in this study demonstrate that there is a high prevalence of anaemia amongst pregnant women in the primary health centre. Given the impact of anaemia on pregnancy outcomes, it is clearly advantageous for clinicians to have a practical and efficient means of screening and treating anaemia in the primary health centre. It is recommended that health promotion and disease prevention campaigns be organised at places of contact with pregnant women and mothers.

It is not only needed to prevent anaemia at hospital levels but also to address the prevailing socio-economic and cultural factors associated with it. The socio-economic status of women should be enhanced in line with the Millennium Development Goals that all the 193 United Nations have agreed to achieve by the year 2015. These goals include eradicating extreme poverty, reducing child mortality rates, improving maternal health, fighting disease epidemics such as AIDS, and developing a global partnership for development. It is hoped that this initiative will help prevent anaemia and enhance pregnancy outcomes.

Multi-centre research is needed to understand the pathogenesis of anaemia amongst pregnant patients from differing cultural backgrounds in primary health centres. The knowledge generated from such research will increase the potential for providing acceptable and appropriate health care to pregnant patients with anaemia.
